# Selecting optimal screening items for delirium: an application of item response theory

**DOI:** 10.1186/1471-2288-13-8

**Published:** 2013-01-22

**Authors:** Frances M Yang, Richard N Jones, Sharon K Inouye, Douglas Tommet, Paul K Crane, James L Rudolph, Long H Ngo, Edward R Marcantonio

**Affiliations:** 1 Harvard Medical School, Beth Israel Deaconess Medical Center, Division of Gerontology, Institute for Aging Research, Hebrew SeniorLife, 1200 Centre Street, Boston, MA 02131, USA; 2 Department of Medicine, Harborview Medical Center, University of Washington, Box 359780, 325 Ninth Avenue, Seattle, WA, 98104, USA; 3 Harvard Medical School, Brigham and Women′s Hospital, Division of Aging, 75 Francis St, Boston, MA, 02151, USA; 4 Harvard Medical School, Beth Israel Deaconess Medical Center, Division of General Medicine and Primary Care, 330 Brookline Ave; CO-230, Boston, MA, 02215, USA; 5 Harvard Medical School, Beth Israel Deaconess Medical Center, Divisions of General Medicine and Primary Care and Gerontology, 330 Brookline Ave, CO-216, Boston, MA, 02215, USA

**Keywords:** Confusion assessment method, Delirium screening, Dimensionality, Factor analysis, Item response theory, Item bank

## Abstract

**Background:**

Delirium (acute confusion), is a common, morbid, and costly complication of acute illness in older adults. Yet, researchers and clinicians lack short, efficient, and sensitive case identification tools for delirium. Though the Confusion Assessment Method (CAM) is the most widely used algorithm for delirium, the existing assessments that operationalize the CAM algorithm may be too long or complicated for routine clinical use. Item response theory (IRT) models help facilitate the development of short screening tools for use in clinical applications or research studies. This study utilizes IRT to identify a reduced set of optimally performing screening indicators for the four CAM features of delirium.

**Methods:**

Older adults were screened for enrollment in a large scale delirium study conducted in Boston-area post-acute facilities (n = 4,598). Trained interviewers conducted a structured delirium assessment that culminated in rating the presence or absence of four features of delirium based on the CAM. A pool of 135 indicators from established cognitive testing and delirium assessment tools were assigned by an expert panel into two indicator sets per CAM feature representing (a) direct interview questions, including cognitive testing, and (b) interviewer observations. We used IRT models to identify the best items to screen for each feature of delirium.

**Results:**

We identified 10 dimensions and chose up to five indicators per dimension. Preference was given to items with peak psychometric information in the latent trait region relevant for screening for delirium. The final set of 48 indicators, derived from 39 items, maintains fidelity to clinical constructs of delirium and maximizes psychometric information relevant for screening.

**Conclusions:**

We identified optimal indicators from a large item pool to screen for delirium. The selected indicators maintain fidelity to clinical constructs of delirium while maximizing psychometric information important for screening. This reduced item set facilitates development of short screening tools suitable for use in clinical applications or research studies. This study represents the first step in the establishment of an item bank for delirium screening with potential questions for clinical researchers to select from and tailor according to their research objectives.

## Background

Delirium is a preventable [1,2] acute confusional disorder. In the US, delirium affects over 2.3 million hospitalized older adults each year [3] at an estimated total annual cost of $152 billion [4]. Recognition of delirium is a prerequisite for developing a coherent treatment program. However, delirium remains under-recognized and is consequently mismanaged in most clinical settings [5].

Formal diagnostic criteria for delirium were first codified in 1980 in the American Psychiatric Association′s Diagnostic and Statistical Manual of Mental Disorders, Version 3 (DSM-III) [6]. Different definitions have appeared in subsequent DSM versions [7-9]. The first appearance of delirium in the International Classification of Diseases occurred in ICD-10 [10]. While the DSM clearly captures the key elements of the delirium syndrome, the DSM criteria themselves can be challenging to apply diagnostically, both in clinical practice and in research settings, particularly for patients who are not communicative [11]. Additionally, the DSM-IV criteria require knowledge of underlying cause before diagnosis can be made. In clinical practice, usually delirium is first recognized and then a search for the underlying cause proceeds. Wide discrepancies in case identification have been reported when different criteria are used [11-13].

There are many methods for research and clinical diagnosis of delirium, operationalizing either the International Classification of Diseases (ICD) or DSM criteria [14]. The most commonly used algorithm for case identification of delirium is the Confusion Assessment Method (CAM) [15]. The CAM reduces the nine original DSM-III-R criteria to four key features, requiring the presence of both 1) acute change in mental status with a fluctuating course and 2) inattention, and either 3) disorganized thinking or 4) altered level of consciousness. A recent comprehensive review showed its strong performance characteristics and widespread use [16]. The CAM algorithm has been used in over 1600 publications over the past 14 years, more than 10 times more frequently than the DSM criteria [16]. The recommended interview prior to completion of the CAM is a short cognitive screening tool, including assessment of attention [17]. However, different researchers may operationalize the CAM features differently. To maximize the accuracy and reliability of the CAM, standardized mental status and neuropsychiatric assessments, questionnaires and ratings should be used to assess delirium symptoms [18]. However, because such assessments may require up to 30 minutes for administration and scoring [18] they are impractical for clinical use and burdensome for research studies. Therefore, reducing the length of screening interviews is an important step in improving case identification. Item response theory is a statistical tool that can help in this process. The goal of our work is to identify the most efficient set of items to determine the presence or absence of each of the CAM features.

Item response theory (IRT) encompasses a set of psychometric tools that—among other things [19]—can help in the selection of optimal test questions to shorten instrument [20-25]. IRT is a statistical framework that relates observed patient data (responses to test items, or diagnostic signs and symptoms) to theoretical (i.e., latent) and presumed continuously distributed constructs. IRT can be considered an extension of classical factor analysis [26] and is a useful tool in test construction because it provides a framework for expressing characteristics of test-takers and test items on a uniform metric. IRT and factor analysis are isomorphic when the factor analysis is performed on a matrix of polychoric correlations and only one latent variable is modeled [26-28]. In this study, the unidimensional factor analysis results are item response theory results, and more globally the multidimensional factor analysis results are multidimensional item response theory [29]. The ordinal dependent variable approach to factor analysis was described by Birnbaum in Lord and Novick′s seminal work on IRT [30], formalized by Christoffersson [31] and Muthén [32].

In our approach, insofar as unidimensionality is an assumption of IRT [33], we sought first to assess the extent to which our data satisfied this assumption before moving on to formal IRT analyses. This feature makes possible the construction of tests for specific uses or specific populations. In many IRT parameter estimation procedures, item parameters are assumed to be fixed and invariant across population subsamples [34]. This is a strength in that tests can be constructed using only some items from a larger bank of items but still produce estimates of person level on the same metric as other tests using different items from the bank.

IRT posits models that express a person′s response (*y*_*ij*_), person-level trait (*θ*_*i*_), and item parameters (*a*_*j*_*,b*_*j*_). Let *y*_*ij*_ represent person *i*′s response to item *j* that is observed as correct (or symptom present) (*y* = 1) or incorrect (or symptom not expressed) (*y* = 0). The probability that a randomly selected person from the population expresses a symptom is

(1) $$P_{j}\left(\theta \right)=P\left(y_{\mathrm{ij}}=1|\theta_{i},a_{j},b_{j}\right)=G\left(a_{j}\left(\theta_{i}-b_{j}\right)\right)$$

where *G* is some cumulative probability transformation, usually the inverse logit, but the normal probability distribution function is also used. The unobserved variable (e.g., latent level for the CAM feature of inattention)θ, is often assumed to be distributed normally with mean zero and unit variance. The difference between a person^′^s latent trait level (*θ*_*i*_) and the item difficulty (or item location, or symptom severity level, *b*_*j*_) defines the probability that a person will display a symptom (e.g., ″Trouble keeping track of what was being said,″ for the CAM feature of inattention). *P*_*j*_(θ) describes the increasing probability of a randomly chosen patient displaying indicator *y*_*j*_ with increasing values of the latent trait *θ*.

If a test symptom severity is greater than the person′s level on the underlying trait or exceeds the test item symptom severity, less likely than not they will express the symptom. The precise probability is modified by the strength of the relationship between the latent trait and the item response, captured with the item discrimination parameter (*a*_*j*_). When logistic regression estimation procedures are used, it is common to include a scaling constant (*D*) so that the logit parameters are standardized [35].

Building tests to suit specific uses can employ the concept of item information [30]. Item information is expressed with *I*_*j*_(*θ*) = *a*_*j*_^2^*P*_*j*_(*θ*)[1 − *P*_*j*_(*θ*)]. The more highly discriminating an item is, the more peaked its information function. Information functions are centered over the item difficulty parameter. Information is analogous to reliability in the sense that it expresses measurement error. Due to the assumption of local independence, item information functions are additive. Local independence is an important basic assumption in IRT along with unidimensionality, where an answer to one item is not contingent or statistically dependent upon an answer to a preceding item. The curve describing the sum of information over the underlying trait is called a test information curve. Taken together, it is possible to achieve fine control over where and how well a given item set measures a latent trait along the latent trait distribution (subject to the availability of items with the desired parameters). The goal of this paper was to identify the shortest set of mental status assessment questions and interviewer observations that could be used to efficiently provide relevant information for screening about a patient′s level on four CAM diagnostic features. We present our approach to developing an item bank for the future development of screening tool using item response theory and related psychometric methods. The context is the future development of predictive tests for distinguishing persons who satisfy each of the four CAM criteria for delirium. Our substantive goal was to develop a parsimonious set of indicators for each of the four key CAM features of delirium to be considered in further developing brief clinically useful screening measures [15].

## Methods

### Design and participants

To meet our objective of identifying a small set of indicators for the core features of delirium that would be useful for screening, we began with a conceptual model of the important symptom dimensions of delirium. This was informed by the CAM [15]—specifically the four features of delirium described above. We identified mental status, neuropsychological performance, and delirium symptom assessment instruments that include specific tests that could provide information relevant to those symptom dimensions. We then identified and obtained an existing data source that included relevant assessment tools. We formed a panel of clinical experts to inform the data handling and statistical and psychometric data analysis. Specific details are described below. The overall construction and evaluation of the item bank is similar to the National Institutes of Health Roadmap Initiative Patient-Reported Outcomes Measurement Information System (PROMIS). The description of the PROMIS psychometric analysis for item banking is found in Reeve et al. [36].

The sample used in this analysis was chosen to provide a high rate of delirium, and was drawn from the screening phase of a randomized controlled trial of a Delirium Abatement Program (DAP) [37]. The trial was conducted in eight post acute care facilities in Massachusetts between years 2000 to 2003. During the enrollment period, 6,354 persons were admitted to one of the eight facilities. All assessments were conducted within three days of admission. Of the 4,744 screened, medical records were unavailable for 92, and 54 were excluded due to coma. The final sample included 4,598 subjects from the screening cohort. Of these 611 (13%) displayed CAM delirium. Patients were only included if they were able to provide assent and their caregivers subsequently provided informed consent. The details of the study have been provided previously by Kiely and colleagues [38]. The Mini-Mental State Examination (MMSE) was used as part of the structured mental status assessment with Delirium Symptom Interview (DSI), Memorial Delirium Assessment Scale (MDAS), and CAM to identify delirium [18]. Review and approval of this study and protocol was provided by the Institutional Review Boards of the Beth Israel Deaconess Medical Center and Hebrew SeniorLife.

### Measurements

Source items, which included direct patient questions, mental status testing, and observational items, were obtained from a structured delirium assessment [18,37], which culminated in a rating of the presence or absence of the four core features of CAM delirium [15]. The DAP trial structured delirium assessment included the Mini-Mental State Examination (MMSE) [39], which assesses orientation to year, season, month, day of the week, date, city or town, name of place, and type of place. The DAP screening assessment also included the Digit Span test [40], which involves asking patients to repeat increasingly long sets of numbers in order forwards and different sets of numbers backwards. The Delirium Symptom Interview (DSI) [41] is a 113-item instrument that includes both questions asked directly of the patient, and a series of structured observations. Assessment areas included attention, organization of thought, level of consciousness, disturbance of perception, sleep and psychomotor activity. The total number of items from the source instruments that were considered in the clinical consensus was 119. The description of the clinical consensus process is reported in detail by Huang and colleagues [42]. All items were dichotomous, as described in Huang et al. [42].

### Statistical analysis

**Figure 1:**
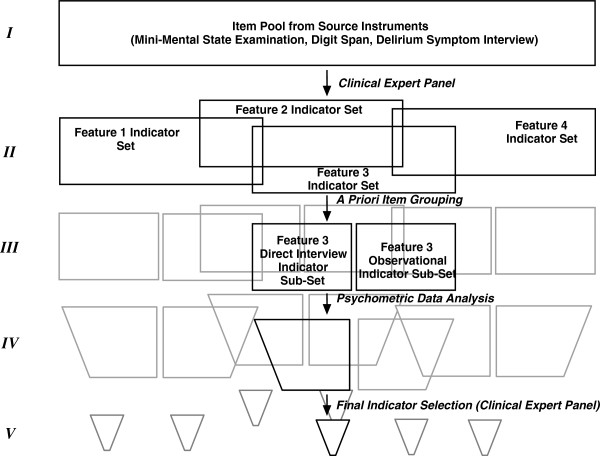
**This figure illustrates the item and indicator selection stage and major process steps.** Stage I begins with source items from established instruments. A Clinical Expert Panel defined indicators for each of four features of delirium, defining indicator sets for each feature (Stage II). Overlapping boxes imply that indicators are not exclusively defined from source items. That is, an item may be used to define an indicator of more than one CAM feature. At Stage III indicators were grouped into sets according to mode of collection of the source item (direct interview vs. observational). Stage IV involved psychometric modeling steps, including dimensionality assessment, factor analysis, and item response theory analyses, resulting in reduction of the indicator set and possible splitting into multiple sub-sets. Stage V indicator sets contain 5 indicators per indicator sub-set as selected by the clinical expert panel.

Our analysis involved multiple stages of item processing and data analysis. We illustrate the stages in Figure 1. The process began with 119 source *items* (data collected from primary assessment instruments) and ended with a reduced set of 103 *indicators* (analytic variables defined from source items), as shown in Table 1.

**Table 1:** Summary of results from dimensionality assessment models

				**Single factor model**			**Two-factor model**		**Bifactor model**	
**Feature**	**Number of proposed indicators**	**Number of modeled indicators**	**Number of significant eigenvalues**	Marginal reliability*	**CFI**^**†**^	**RMSEA**^**‡**^	**CFI**	**RMSEA**	**Large secondary loadings**	**Retained dimensions**
Feature 1-Acute Change and Fluctuating Course-*Direct Interview*	17	15	1	0.32	0.94	0.03	0.98	0.02	n/a	1
Feature 1-Acute Change and Fluctuating Course-*Observational*	12	11	1	0.81	0.99	0.05	n/a	n/a	n/a	1
Feature 2-Inattention-*Direct Interview*	15	15	2	0.80	0.96	0.06	0.99	0.03	no	2
Featured 2-Inattention-*Observation*	8	8	1	0.72	0.98	0.04	n/a	n/a	n/a	1
Featured 3-Disorganized Thinking-*Direct Interview*	13	13	2	0.64	0.98	0.05	0.99	0.03	no	1
Featured 3-Disorganized Thinking-*Observational*	13	13	2	0.89	0.90	0.08	0.98	0.04	yes	2
Featured 4-Altered Level of Consciousness-*Observational*^*§*^	25	14	2	0.94	0.89	0.05	0.98	0.03	yes	2

Note: *Marginal reliability coefficient is based on each feature at *θ*_50_^+^, ^†^Comparative Fit Index, ^‡^Root Mean Squared Error of Approximation. n/a implies the result is not applicable because the relevant models were not estimated in a bi-factor model. ^§^Feature 4 –Altered Level of Consciousness-Direct Interview was dropped because items were redundant with Feature 2—Inattention-Direct Interview.

#### Expert panel review

Our Clinical Expert Panel (CEP) consisted of one geriatric psychiatrist, one geriatric nurse, one behavioral neurologist, one neuropsychologist, and three internists/geriatricians, all of whom were experts in delirium assessment and familiar with the CAM algorithm. Details regarding the CEP review process are described elsewhere [42]. Briefly, we summarize the stages of CEP review process most relevant to this study. Stage I began with identifying source items from established instruments. The CEP classified *indicators* from source *items* according to relevance for each of four features of delirium as defined by the CAM algorithm (Stage II). Indicators were then sub-classified as reflecting observational data (i.e., a rating of a symptom observed by trained interviewer) *versus* direct interview data (i.e., a verbatim response to a directly asked question, including cognitive test questions) (Stage II). *Items* could be assigned to more than one feature, as implied by the overlapping boxes in Figure 1. For example, the first orientation question ″What is the year?″ was assigned to both CAM Feature 2, Inattention and CAM Feature 3, Disorganized thinking.

#### Exploratory data analysis

At Stage III, eight indicator sets were defined (i.e., indicator sets assigned to each of the four CAM features, separately considering direct interview and observational indicators). We performed exploratory data analysis within indicator sets, including cross-tabulations and data quality assessment (e.g., missing data checking. Item cross-tabulations were carefully examined for voids (empty cells) that might arise from logically dependent response sets. For example, a pair of items with a logical dependency could be an item that (a) assesses whether symptom *x* was present followed by an item (b) that assesses the symptom frequency. Linear modeling of such dependent items is not appropriate. When we found logical dependencies, the expert panel restructured the indicators. For example, generating a single ordinal composite to summarize both presence and frequency of a symptom.

We performed an empirical multi-collinearity check for local dependency among pairs of indicators for which correlations could be not be estimated. We fit a weighted least-squares factor analysis model using Mplus software (version 5.2, Muthén & Muthén, Los Angeles CA) [43] to the indicator sets and examined error messages indicating sparsely populated cells, potentially due to logical dependencies. We developed an automated iterative algorithm to rank individual indicators according to the number of times correlations could not be estimated, and we dropped the indicators most frequently involved. Every indicator dropped was reviewed by the CEP. The CEP recommended that some indicators should be retained if the symptom was clinically important. We then repeated the empirical multicollinearity checking but forcing the algorithm to retain the flagged items.

#### Assessment of dimensionality

Within each indicator set, we evaluated the assumption of unidimensionality using permuted parallel analysis [44] and factor analysis (exploratory, confirmatory, and bi-factor [45] models). Parallel analysis involves comparing observed and random eigenvalues. Eigenvalues, or latent roots, of a correlation matrix can be interpreted as variances for (latent) variables derived from the observed variables [46]. Random eigenvalues were estimated empirically by randomly assigning indicators to persons and extracting eigenvalues from the resulting correlation matrix. With multiple replications of the random data, this procedure represents a permutation test on the observed eigenvalues. We defined significant eigenvalues as those where the observed eigenvalue exceeds the 97.5^th^ percentile of eigenvalues from the permuted (random) data.

We used the number of significant eigenvalues, *m*, to set the number of factors to examine in an exploratory factor analysis (EFA). Based on the results of the EFA we specified a simple structure confirmatory factor analysis (CFA) [47] and bi-factor analysis (BFA) [45] models. We assigned each item to a single factor in CFA—or to a specific factor in BFA—on which it had the largest loading. For example, if the parallel analysis suggested the presence of two significant factors, we examined the EFA solution for two factors. A simple structure CFA model would have two factors, and each indicator would load on the factor for which it had the highest factor loading in the EFA solution. A BFA model would use this same factor loading pattern, but would include a general factor loading on all indicators, and factor correlations would be constrained to zero.

We considered the preponderance of the evidence in making dimensionality decisions, together with the input of the CEP on the interpretation of secondary factors. Evidence of sufficient unidimensionality included only one significant factor on permuted parallel analysis. Signs of failure of the indicator set to conform to unidimensionality included significant eigenvalues beyond the first, improvement in fit statistics [the confirmatory fit index (CFI) [48] and root mean square error of approximation (RMSEA) [49]], between a single factor model and an *m*-factor CFA model, and greater factor loadings on specific factors relative to loadings on general factors in the BFA. When we rejected the assumption of unidimensionality, the indicator sets were split into *m* sub-sets to achieve sufficiently unidimensional indicator sets for IRT-based data analyses.

#### Item analysis

**Figure 2:**
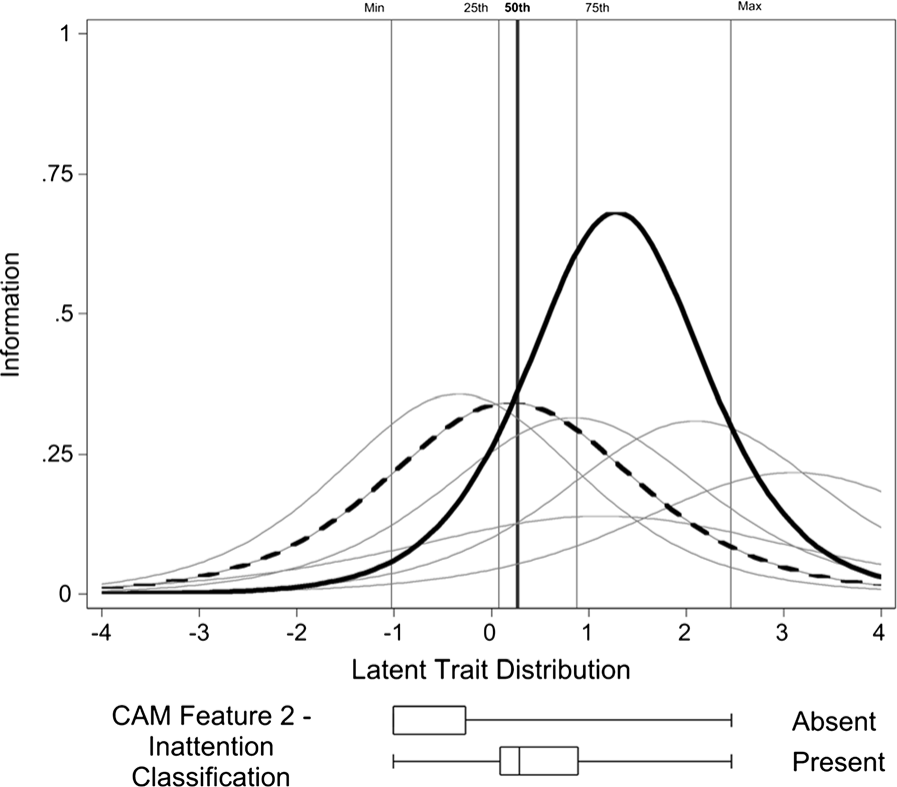
**This figure plots the item information curves for all direct interview based indicators identified by the clinical consensus panel as measures of CAM feature 2 -*inattention.*** Parameters were estimated from analysis of delirium screening data for 4,598 persons evaluated for inclusion in the DAP Trial [38]. Two lines are highlighted: *″List the months of the year backwards.″* (heavy dotted line) and *″List the days of the week backwards.″* (solid bolded line). Box and whisker plots show distributions of estimates of latent trait scores for participants classified as CAM feature 2 - inattention positive and negative by the CAM algorithm (see text for details). Vertical reference lines for key percentiles of the feature positive group are illustrated in the main panel. Note that the whiskers identify the minimum and maximum in this group. Among all the indicators evaluated, the two highlighted indicators have the maximum information content at the 50th percentile of the population of patients who had Feature 2, Inattention, coded as present, and therefore represent indicators with optimal screening efficiency for the presence of inattention.

We then analyzed items based on item response theory (IRT). Our goal was to identify indicators that provide high information content, found at the peak of the curve (Figure 2), for the latent trait presumed to cause the observed responses (*θ*_*i*_) in a region that would be relevant for screening purposes. To identify this region of *θ* item information functions were used together with CAM feature positive rating information in the source data set. For each unidimensional indicator set evaluated we estimated item parameters and computed *expected a posteriori* (EAP) [50] estimates of the evaluee′s underlying latent trait level $\left(\hat{\theta }\right)$. We then identified the 50^th^ percentile of this $\hat{\theta }$ distribution among evaluees who satisfied criteria for the particular CAM feature. We chose items for retention in the reduced item set that maximized item information *I*(*θ*) at this level of *θ.* The rationale for this step is as follows. Severe symptoms, such as agitation or perceptual disturbances, may be clear signs of delirium, but are rare in the population. Even if these severe symptoms are highly correlated with *θ,* they represent poor items for a screening test because they have a low base rate and do not provide information about where most people are. More sensitive screening items would be items that are more prevalent among persons ultimately assigned to the CAM feature positive condition (i.e., near the 50^th^ percentile), but not so uncommon that only the most severe cases of delirium demonstrate such signs and symptoms. We performed sensitivity analyses evaluating ranks of item information content at the 25^th^ and 75^th^ percentiles of the CAM feature positive sub-group, and identified essentially the same indicator sets. We calculated the marginal reliability of the unidimensional traits at the level of the latent trait corresponding to the median estimated level among patients rated as CAM feature positive.

All eigenvalues, EFA, CFA, and IRT models were estimated with Mplus software (version 5.2, Muthén & Muthén, Los Angeles CA) using limited information weighted least squares estimation with mean and variance adjustment [43,51,52]. Permuted parallel analyses were performed with a custom Stata macro (version 10, Stata Corp, College Station, TX). Bi-factor models were estimated with Gibbons and Hedeker′s BIFACTOR software (Center for Health Statistics, University of Illinois at Chicago). Item characteristic curves and information functions were calculated using formulae provided in Baker and Kim [53] and Thissen and Wainer [54] and plotted using Stata. All syntax and command files and custom software are available upon request (RNJ).

#### Final indicator selection

The final results of our analytic steps (Stage V, Figure 1) represent the selection of up to 5 indicators per CAM feature. The reason for choosing 5 indicators is because studies have shown more than four indicators per latent trait are ideal for arriving at a proper solution in latent variable modeling, and diminishing returns are observed with more than five indicators [55,56].

## Results

Participant characteristics are summarized in Table 2. The mean age was greater than >80 years. Women represented over two thirds of the sample. Baseline cognitive impairment was common: the mean Mini-Mental Status Examination (MMSE) score was 21.4 (Standard Deviation, SD ± 6.3). CAM Delirium was present in about 1 in 8 of the sample.

**Table 2:** Characteristics of study participants

	**Mean**	**(SD)**	**Observed**
**Characteristic**	**or n**	**or (%)**	**range**
Total [n (%)]	4598	(100)	
Age [M (SD)]	81.5	(7.7)	[64.0–104.0]
Sex [n (%)]			
Male	1425	(31.0)	
Female	3172	(69.0)	
Race/Ethnicity [n (%)]			
White	3918	(85.2)	
Black/African American	269	(5.9)	
Other races	29	(0.6)	
Missing	382	(8.3)	
Delirium Present [n (%)]	611	(13.3)	
Mini‐Mental State Examination Score [M (SD)] (scored 0–30, 30 best)	21.4	(6.3)	[0.0–30.0]
Mini‐Mental State Examination Score group [n (%)]			
Severe cognitive impairment (0–17)	1018	(22.1)	
Cognitive impairment (18–23)	1560	(33.9)	
No cognitive impairment (24–30)	2019	(43.9)	

Note: The race/ethnicity information was collected at screening and based on nursing home and medical records, which had missing or incomplete data for race/ethnicity.

The clinical expert panel defined CAM feature *indicators* from source *items* drawn from the MMSE orientation items, digit span, and DSI*.* We analyzed the resulting 135 indicators following the psychometric modeling steps described in the methods (multi-collinearity checking, dimensionality determination, IRT). Results are summarized in Table 1. This table lists by CAM feature (column 1) the number of indicators proposed by the clinical expert panel (column 2), the number of indicators remaining after empirical multi-collinearity checking (column 3), number of significant eigenvalues following permuted parallel analysis (column 4), and the marginal reliability of each feature at *θ*_50_^+^ (column 5). Columns 6–7 summarize model fit statistics and estimates of a single factor model fit to the indicator set, and columns 8 and 9 the model fit statistics for the *m*-dimensional model. As indicated in Table 1, no indicator set had more than two significant eigenvalues based on the permuted parallel analysis. Column 10 summarizes whether large secondary loadings were observed (secondary factor loading exceeded the common factor loading for a given item) in the BFA. Column 11 reports the final adjudication of the expert panel on the number of retained dimensions. Three indicator sets identified more than one secondary factor, and the expert panel agreed with the results. When only one significant eigenvalues was detected, model fit statistics were generally good (CFIs > 0.94 and RMSEAs < 0.05) [57].

The next step was to identify items that provided high information content in a region of the underlying trait assessed by the items. We did this by evaluating the item information at the 50^th^ percentile of the latent trait distribution underlying the indicator set (or sub-set) among those participants who were rated as CAM feature positive. We identify this level of the latent trait as the 50^th^ percentile (*θ*_50_^+^) curve. An example of one such curve is shown in Figure 2. This figure plots item information curves for the indicators identified by the Clinical Expert Panel as measures of CAM feature 2 – *inattention—direct interview*. All indicators are illustrated, but we highlight two for discussion: *″List the months of the year backward″* (heavy dotted line) and *″List the days of the week backward″* (solid bolded line). The box and whisker plots beneath the horizontal axis indicate the distributions of posterior estimates of latent trait scores for participants ultimately classified as CAM feature 2 - *inattention* positive and negative. Vertical reference lines for key percentiles of the CAM feature positive group are illustrated in the main panel.

This figure illustrates several important points about the analysis of this indicator set. First, the latent trait distributions for the CAM feature positive and negative sub-groups show wide separation. Nevertheless, most of the item difficulty parameters (located where the information functions peak) are above the 75th percentile of the CAM feature positive group. Thus, most of indicators in this set contribute the most information at very severe levels of the underlying trait. Such items would not be useful for screening purposes, even if the assessed symptoms were pathognomonic of delirium. Our goal is to derive a test information curve tuned for screening purposes. We approach this by choosing the items with the most information at the 50^th^ percentile for our item bank. The two highlighted items provide the most information at the 50^th^ percentile of the latent trait distribution in the feature positive group. This is the area of the latent trait of greatest interest for screening purposes.

The top 5 delirium indicators ranked in order of information at the 50^th^ percentile of the latent trait distribution for the CAM feature positive subgroup are displayed in Table 3. The tabulated indicators comprise 39 original assessment items. In Table 3 we also present the item information (*a*) and difficulty (*b*) parameters for each indicator.

**Table 3:** Source items and indicator IRT parameters for top five indicators identified for each dimension of each CAM feature*

**Feature and Indicator Selection (top five indicators)**	**Discrimination**	**Difficulty**
**threshold level*(θ***_**50**_^**+**^**) on latent trait**	***(a)***	***(b)***
*Feature 1 -Acute Change and Fluctuating Course- Direct interview* (*θ*_50_^+^ = − 0.20)		
Felt confused during the past day	0.96	1.72
Thought you were not really in (name of facility)	1.00	2.21
Saw things that were not really there	1.33	2.29
Thought things were moving that were not really moving	0.98	2.66
Heard things that were not really there	1.55	2.56
*Feature 1 -Acute Change and Fluctuating Course-Observational* (*θ*_50_^+^ = 1.17)		
Level of consciousness fluctuated	2.97	1.80
Level of attention fluctuated	1.83	1.46
Speech/thinking fluctuated	1.98	1.77
Evidence of disturbance of sleep	1.97	1.83
Psychomotor activity fluctuated	1.57	2.43
*Feature 2 -Inattention- Direct interviewFirst Factor* (*θ*_50_^+^ = 0.22)		
What is the year? ^†^	1.57	1.14
What is the month? ^†^	1.86	1.17
What is the day of the week? ^†^	1.21	0.78
What type of place is this? ^†^	1.55	1.23
What is the name of this place? ^†^	1.12	0.24
*Second Factor* (*θ*_50_^+^ = 0.27)		
Days of the week backwards	1.65	1.29
Months of the year backwards	1.17	0.20
Digit span backwards 3 Numbers ^‡^	1.12	0.85
Digit span backwards 4 Numbers ^‡^	1.20	−0.34
Digit span forwards 4 Numbers ^‡†^	1.11	2.09
*Feature 2 -Inattention- Observational* (*θ*_50_^+^ = 0.38)		
Trouble keeping track of what was being said	1.26	0.25
Level of attention fluctuated	1.74	1.55
Unaware of environment	2.09	1.91
Distracted by environmental stimuli	1.28	2.06
Staring into space	1.09	2.11
*Feature 3 -Disorganized Thinking Direct interview* (*θ*_50_^+^ = 0.67)		
What type of place is this?^†^	1.56	1.23
What is the year? ^†^	1.49	1.17
What is the month? ^†^	1.74	1.20
What is the day of the week? ^†^	1.20	0.79
What is the name of this place? ^†^	1.11	0.24
*Feature 3 -Disorganized Thinking Observational*^*§*^*First Factor* (*θ*_50_^+^ = 1.03)		
Unclear or illogical flow of ideas	2.07	1.29
Changes the subject suddenly	1.83	1.90
Conversation was rambling	1.36	1.68
Words or phrases that were disjointed or inappropriate	1.33	2.21
Speech/thinking fluctuated	1.17	2.27
*Feature 4 -Fluctuating Course and Altered Level of Consciousness- ObservationalFirst Factor* (*θ*_50_^+^ = 1.99)		
Sleepy, or stuporous, or comatose	9.70	1.70
Disturbance of sleep	3.18	1.81
Lethargy and sluggishness	1.41	1.44
Slowness of motor response	1.23	1.70
Expressed a paucity of thoughts	0.97	3.23
*Second Factor* (*θ*_50_^+^ = − 0.14)		
Restlessness	1.44	2.02
Speech unusually fast or pressured	0.74	3.71
Excessive absorption with ordinary objects	2.31	2.29
Increased speed of motor response	0.69	4.49
Grasping/picking	2.68	2.20

Note: All items from the Delirium Symptom Interview [41] except those noted with † which are derived from orientation items [39,58] and ‡ which derive from the digit span test [40] § - The second factor with 2 additional items (3 indicators) that were identified for Feature 3 was not evaluated using IRT methods. The threshold level is the estimated median on the level on the underlying latent trait for persons CAM feature positive ((*θ*_50_^+^)). *Top 5 items with most information at *θ*_50_^+^.

Of note, we did not pursue IRT modeling for the second observational factor of Feature 3 (*disorganized thinking*) because only three items loaded on this factor: limited speech, paucity of thoughts, and slow speech. We also did not include the direct interview items of Feature 4 (*altered level of consciousness*) because the item set was redundant with Feature 2 (*inattention-direct interview*). For Feature 4 (*altered level of consciousness-observational*), the second factor showed all items having very low information content at the 50^th^ percentile, so for this feature, we made our decision based on the 75^th^ percentile in the CAM feature positive group.

The marginal reliability estimates for each of the CAM IRT-derived features are shown in Table 1. The marginal reliability estimates are based on the mean standard error of the IRT scores for the items at the 50^th^ percentile of the latent trait distribution for the CAM feature positive group. Most marginal reliability estimates were 0.80 or higher, with higher reliability approaching a coefficient of 1, suggesting good reliability at the area of reliability relevant to screening.

## Discussion

Through an iterative process pairing a clinical expert panel with psychometric data analysis, we have identified a set of 48 indicators, derived from 39 items that are optimal for screening patients for the four core features of delirium as defined in the CAM algorithm. The symptoms assessed are clinically relevant and optimize psychometric properties for screening. The resulting item pool can be used to develop short form screening instruments for clinical or research use.

A challenge we faced in our item selection procedure is what criteria to use for selecting candidate items that would be optimal for screening. To this end, we generated item information functions for each indicator, and selected indicators that maximized information around the median underlying latent trait level for persons with each CAM feature positive. Some items, even those that are pathognomonic for a particular CAM feature, may have been omitted if they provide most of their information around a level of severity that is not relevant for screening. Our approach leads to measures that maximize measurement precision of underlying latent traits at a level that is important for separating persons who are or who are not classified as demonstrating the CAM feature.

Our goal was to define a set of items for clinical researchers to construct a short form for the routine screening of delirium to replace lengthy batteries of mental status, neuropsychological assessment, and observational items. The significance of this work is for the future establishment of validated instrument for delirium screening. Our work represents a first step in development of a more refined delirium screening instrument. The approach used here may be more widely applicable to a broad array of conditions that rely on multi-item assessment batteries to screen for delirium. The innovation of the approach we used in this study is the use of IRT to select optimal items for screening that maximizes psychometric information at the latent trait level that discriminates between persons who do and do not demonstrate the four core features of delirium described in the CAM algorithm. The items were chosen in an iterative fashion that incorporates an interdisciplinary perspective from both clinical and methodological expertise in measurement research. The novel approach used in this study for case identification in delirium allows the interdisciplinary team to select items based on item information at the 50^th^ percentile for those who screen positive on the specific CAM feature. Ideally, in the near future our analysis will be enhanced by computer assisted bedside interviewing with well characterized item banks and adaptive testing algorithms tuned to distinct purposes (e.g., grading delirium severity, screening for probable delirium).

Several caveats are worthy of discussion. First, our study involved a single, albeit very large, sample of acutely ill elderly patients. Future work will be needed to extrapolate our findings in other samples. Second, the operationalization of the critical theta value for screening could have been incorrect; however, we performed sensitivity analyses demonstrating that using values other than the median among CAM feature positive persons identified similar items. Third, any delirium tool developed from the identified items would need to be validated in an independent cohort. We are actively pursuing this work.

The DSM-IV and ICD-10 are used for diagnosis and coding by trained clinicians. In contrast, the design and purpose of the current study was to identify items for delirium screening based on the four CAM features, which can be done by both clinicians and trained non-clinicians. Therefore, this research may not directly inform diagnosis relying only and strictly on the DSM and ICD.

Another limitation of our analysis is that age, sex and race/ethnicity, have not been considered in this analysis. These factors have been shown to be associated with the differential expression of signs and symptoms in other psychiatric and cognitive disorders, although not necessarily in delirium. Our results assume that the measurement of symptoms of CAM features is invariant across major sociodemographic groups. A future direction for potentially improving the current instrument is to examine measurement bias due to age and gender.

## Conclusion

We have identified a candidate set of delirium indicators for the future development of a short assessment for detecting delirium. In a health care setting where time and resources are limited, accurate and brief assessments are greatly needed for systematic case-finding of delirium. In research settings, efficient assessment is crucial to reduce participant burden and maximize validity. This study lays the groundwork for the development of short forms for a variety of clinical conditions. Future work is needed to further apply this methodology to develop short form tools for delirium detection and research application, and to validate these new instruments across a broad range of populations and settings.

Presented at the Gerontological Society of America 64^th^ Annual Meeting in Boston, Massachusetts, USA.

## Competing interests

The authors declare that they have no competing interests.

## Authors’ contribution

FMY participated in the design, analysis, and drafted the article. RNJ participated in the acquisition of data, conception of design and analysis, and drafted the article. SKI participated in the design and analysis and provided critical revision of the manuscript. DT participated in the analysis and critical revision of the manuscript draft. PKC participated in the conception of the design and analysis and provided critical revision of the manuscript. JLR participated in the acquisition of data and provided critical revision of the manuscript. LHN participated in the analysis and provided critical revision of the manuscript. ERM obtained support for the research, participated in the acquisition of data, contributed to the design and analysis and provided critical revision of the manuscript. All authors read and approved the final manuscript.

## Funding sources

This work was funded in part by grants from the National Institute on Aging: R01AG030618 (ERM), K24 AG035075 (ERM), R03AG025262 (RNJ), R01AG17649 (ERM), and P01AG031720 (SKI). Dr. Inouye is supported by the Milton and Shirley F. Levy Family Chair.

## Pre-publication history

The pre-publication history for this paper can be accessed here:

http://www.biomedcentral.com/1471-2288/13/8/prepub
